# Antiviral lectin Q-Griffithsin suppresses fungal infection in murine models of vaginal candidiasis

**DOI:** 10.3389/fcimb.2022.976033

**Published:** 2022-10-18

**Authors:** Henry W. Nabeta, Amanda B. Lasnik, Joshua L. Fuqua, Lin Wang, Lisa C. Rohan, Kenneth E. Palmer

**Affiliations:** ^1^ Department of Microbiology and Immunology, School of Medicine, University of Louisville, Louisville KY, United States; ^2^ Center for Predictive Medicine for Biodefense and Emerging Infectious Diseases, University of Louisville, Louisville KY, United States; ^3^ Department of Pharmacology and Toxicology, School of Medicine, University of Louisville, Louisville KY, United States; ^4^ Infectious Diseases, Magee-Womens Research Institute, Pittsburgh, PA, United States; ^5^ Department of Obstetrics, Gynecology, & Reproductive Sciences, School of Medicine, University of Pittsburgh, Pittsburgh, PA, United States; ^6^ Department of Pharmaceutical Sciences, School of Pharmacy, University of Pittsburgh, PA, United States

**Keywords:** Griffithsin, Q-Griffithsin, antifungal, candidiasis, vaginal, lectins

## Abstract

Resistance to antifungal agents in vulvovaginal candidiasis has resulted in increasing morbidity among women globally. It is therefore crucial that new antimycotic agents are developed to counter this rising challenge. Q-Griffithsin (Q-GRFT) is a red algal lectin, manufactured in *Nicotiana benthamiana*. Griffithsin has well characterized broad spectrum antiviral activity and has demonstrated potent *in vitro* activity against multiple strains of *Candida*, including *C. albicans*. We have been working to incorporate Q-GRFT into topical microbicide products to prevent HIV-1 and HSV-2 transmission. The goal of this study was to evaluate the efficacy of a prototype Q-GRFT dosage form in prophylactic and therapeutic murine models of vaginal candidiasis, through microbiologic, histopathologic, and immune studies. In a preventive model, in comparison with infected controls, Q-GRFT treatment resulted in a lower fungal burden but did not alter the number of vaginal neutrophils and monocytes. In a therapeutic model, Q-GRFT enhanced fungal clearance when compared with infected untreated controls. Finally, histopathology demonstrated lower vaginal colonization with *C. albicans* following Q-GRFT treatment. Our results demonstrate that Q-GRFT has significant preventive and therapeutic activity in vaginal candidiasis offering additional benefit as a topical microbicide for prevention of HIV-1 and HSV-2 transmission.

## Introduction

Vulvovaginal candidiasis (VVC) is an ongoing global challenge, and is predominantly caused by the common fungal pathogen, *Candida albicans* (Sobel, 1985). Approximately 75% of women will develop at least one episode of vulvovaginal candidiasis during their lifetime (Sobel, 2007). About 138 million women globally are affected by recurrent vulvovaginal candidiasis (RVVC) annually, with the numbers expected to increase to 158 million each year, by 2030 (Denning et al., 2018). *Candida* organisms are commensals in the vagina, with overgrowth resulting in vaginal and vulval inflammation contributing to the pathological hallmarks of infection (Fidel et al., 2004). Factors that predispose women to candidiasis include use of oral contraceptives, hormone replacement therapy, pregnancy and antibiotic use (Rivers et al., 2011; Fischer and Bradford, 2011). Recurrent vaginal *Candida* infections are associated with mental discomfort (Sobel, 2016), in addition to the physical symptoms that include pruritus, burning pain, profuse leucorrhea, redness and interrupted and restless sleep as a result of vulval and vaginal mucosal irritation (Peters et al., 2014; Qu et al., 2019).

The major drugs currently used for the treatment of vaginal candidiasis include azoles, echinocandins, and polyenes. Unfortunately, there are increasing reports of resistance by fungal pathogens to these antifungals (Baddley et al., 2008; Alexander et al., 2013; Toda et al., 2019). The resistance is attributed to the static function of many antifungals, in addition to microbial recalcitrance upon repeated drug exposure (Sobel et al., 2003; Pappas et al., 2016). In addition, long-term drug use, prophylactic administration, and exposure to antifungals through agriculture and contaminated food consumption contribute to the growing trend of drug resistance (e Silva and da Costa, 2012; Verweij et al., 2016; Brauer et al., 2019). Furthermore, intrinsic natural resistance to antifungal therapy has been demonstrated in some pathogenic fungal species including azole-resistant *Aspergillus* species (Leonardelli et al., 2016), fluconazole-resistant *C. krusei* (Sanguinetti et al., 2015) and *C. glabrata* (Morio et al., 2017), and echinocandin- resistant *Cryptococcus neoformans*. This demonstrates the urgent need to develop more antifungal agents and strategies relevant to the eradication of these infections.

The vaginal mucosa is the first line of defense against *Candida* through maintaining an acidic mucosal pH that is not optimal for *Candida* and providing anatomical and physiological barriers to infection (Diamond et al., 2008; Moyes and Naglik, 2011). Previous research by numerous groups has demonstrated that *Candida* overgrowth triggers an epithelial cell-mediated cytokine response, with a resultant recruitment of immune cells like neutrophils, dendritic cells, and T cells (Weindl et al., 2007; Moyes and Naglik, 2011; Zhang et al., 2018). Additionally, symptomatic infection demonstrates elevated cellular infiltration with PMNs and variable fungal presence, whereas protection from VVC has been associated with limited or absent inflammatory responses in the vagina (De Luca et al., 2013). However, recently, vaginal candidiasis has been determined to demonstrate a hallmark immunopathogenesis that involves an influx of neutrophils and pro-inflammatory cytokines associated with the inflammasome, and a dysfunction of the neutrophils. This results in a chronic inflammatory condition with no observable clearance of *Candida* (Roselletti et al., 2017; Willems et al., 2020).

Numerous endogenous and plant-derived lectins have previously demonstrated *in vitro* antifungal activity (Gomes et al., 2012; Wang et al., 2013). Griffithsin (GRFT) is a lectin originally derived from red alga *Griffithsia* sp. GRFT has demonstrated broad-spectrum antiviral properties and activity (Mori et al., 2005; O'Keefe et al., 2010; Lo et al., 2020). In previously published research, we showed that Griffithsin has no cellular cytotoxicity at the concentration/dose tested; does not induce production of inflammatory cytokines when exposed to cultured human cells or tissue explants; and is safe when administered to laboratory animals (O'Keefe et al., 2009; Kouokam et al., 2011; Nixon et al., 2013; Barton et al., 2014; Barton et al., 2016; Kouokam et al., 2016). Native GRFT is prone to oxidation (Kramzer et al., 2021), and our group has developed an engineered form, Griffithsin-M78Q (Q-GRFT), with improved stability, and similar antiviral activity to GRFT. We and others have demonstrated that GRFT and Q-GRFT are both safe and efficacious in preclinical models of HIV-1 and HSV-2 infection (O'Keefe et al., 2009; Kouokam et al., 2011; Férir et al., 2011; Nixon et al., 2013; Kouokam et al., 2016; Derby et al., 2018; Girard et al., 2018; Günaydın et al., 2019; Yang et al., 2019; Tyo et al., 2020; Tyo et al., 2020; Kramzer et al., 2021), as well as in early-stage human clinical studies (Teleshova et al., 2022). Recently, we reported a novel antifungal activity of Q-GRFT, with potent growth inhibition of *Candida* species of human importance including *C. albicans*, *C. parapsilosis*, *C. krusei*, *C. glabrata* and against strains of the pan-resistant *C. auris* (Nabeta et al., 2021). It is possible the Q-GRFT impacts other members of the human microbiome, although those data to our knowledge, are not yet available in literature. Additionally, our *in vitro* studies have suggested that Q-GRFT binds to α-mannan *in C. albicans’* cell wall, impairs membrane barrier integrity and likely induces reactive oxygen species formation, with resultant damage to intracellular organelles. Compared to PBS-control, Q-GRFT treatment impaired normal *Candida* cell division, with fungal cells demonstrating failed attempts at budding. Q-GRFT treated cells were circular, with a wrinkled and desiccated rough appearance, with multiple bud scars and loss of polarity. Contrastingly, PBS-treated cells were normally shaped (ovoid), with smooth surfaces and normal budding polarity. Moreover, Q-GRFT induced the expression of genes required to counter cell stress and sustain survival (Nabeta et al., 2021). To our knowledge, no study has reported the efficacy of Q-GRFT in vaginal candidiasis *in vivo* models. Here, we investigated the efficacy of Q-GRFT in vaginal candidiasis using prophylactic and therapeutic murine models. We describe the impact of topical Q-GRFT administration on vaginal fungal burden and the immunological consequence of *C. albicans* infection in the context of topical Q-GRFT therapy.

## Materials and methods

### Mice

Female CBA/J mice (Jackson Laboratories), aged 6-8 weeks were maintained under specific pathogen-free conditions in the Clinical and Translational Research Building vivarium, at the University of Louisville, Louisville, Kentucky. Experiments were performed after animals were acclimated to vivarium conditions for at least one week. In the preventive/prophylactic model, N= 10 mice per group were used for the experiments, with 6 groups employed to test our hypothesis. In the therapeutic model, N=20 mice per group were used for the experiments, with 6 groups employed to test our hypothesis.

### *Candida albicans* and vaginal inoculation

The *C. albicans* ATCC 32032 strain was grown on Sabourand dextrose agar plates overnight at 30°C prior to use, and cell preparation done with slight modifications to the animal model development protocol by Conti et al. (2014). Briefly, 10 milliliters of Sabourand dextrose media were inoculated with 1 colony of *C. albicans* from the agar plate and incubated at 30°C with shaking for 18 hours. Cells were then sub-cultured 1:100 dilution overnight, followed by preparation of 1.0 × 10^8^ cells/mL blastospores from the stationary phase, that were suspended in sterile PBS. Cells were kept on ice until when vaginal inoculation was performed in mice. Twenty microliters of the *C. albicans* preparation were dispensed into each mouse’s vagina using a P50 positive displacement pipettor.

### Estradiol treatment, lavage, and fungal burden

Estradiol (SIGMA Life Science, Lot# BCBW5905) was dissolved in sesame oil (SIGMA, Lot# MKCG9353) to a concentration of 0.5 mg/mL. Mice were then injected subcutaneously with 100 µL of the hormonal preparation in the lower abdomen 3 days prior to *C. albicans* challenge, and then once weekly for the duration of the experiment. To perform the lavage, 100 µL of sterile PBS were dispensed into the mouse vagina and aspirated back and forth several times, and then transferred to labelled Eppendorf tubes on ice. The lavage was then diluted 1:100, and 50 µL of the diluted fluid plated on Sabourand agar. Colli rollers were used to spread the lavage. The plates were incubated at 30°C for 24-48 hours, and colonies counted to establish the fungal burden.

### Vaginal treatment

Forty microliters (40 µL) of a 1% Q-GRFT gel formulated in Carbopol (400 ng), 40 µL of Carbopol placebo gel, 100 µL of nystatin solution at a concentration of 20 mg/mL (Mayne Pharma, Greenville, NC, USA) and 100 µL of sterile 1X PBS were instilled per vaginum in mice from the different animal groups, using appropriate pipettors.

### Hematoxylin and Eosin, periodic acid Schiff staining

For Hematoxylin and Eosin (H & E) staining, sections were deparaffinized and placed in xylene. Sections were then hydrated in alcohol and water baths and stained in hematoxylin for 3 minutes. They were then washed in running water for 5 minutes, differentiated in 1% acid alcohol for 5 minutes, washing in running tap water, dipped in alkaline solution (ammonia water) and washed again. They were subsequently stained in 1% Eosin Y for 10 minutes, washed in tap water for 3 minutes, dehydrated in increasing concentrations of alcohols and then cleared in xylene. Sections were then mounted and observed under a microscope.

For periodic acid Schiff (PAS) staining, sections were dewaxed followed by incubated in 0.5% periodic acid for 5 minutes, washed in running tap water for 3 minutes and then immersed in Schiff’s reagent for 15 minutes. Sections were then washed in tap water for 5 minutes, counterstained with hematoxylin for 2 minutes, washed in running tap water for 3 minutes, dehydrated in ethanol and cleared in xylene for 5 minutes. Sections were then mounted with Entellan^®^ and a cover slip applied. The sections were then viewed under a microscope.

### Flow cytometry analysis

Cellular phenotypic analysis was carried out using flow cytometry with the following antibodies: CD45 (Fisher Scientific catalog# BDB564590, BD Biosciences, San Diego California) and CD11b (Fisher Scientific catalog# BDB557686, BD Biosciences, San Diego California), and F480 (Catalog# 123110, BioLegend, San Diego, California), Ly6G (Fisher Scientific catalog# BDB566435, BD Biosciences, San Diego California), and viability dye (Fisher Scientific catalog# BD565388, BD Biosciences, San Diego California). Vaginal lavage specimens were added to complete RPMI medium (Fisher Scientific catalog# SLM140B, MilliporeSigma™) supplemented with 1M HEPES, penicillin/streptomycin, fetal bovine serum (Catalog# BDB554656) and 2-Mercaptoethanol, filtered and centrifuged for 5 minutes at 1600 rpm. One million cells were then added to appropriate flow cytometry tubes, followed by washing with FACS buffer (Fisher Scientific catalog# BDB5544656, BD Biosciences, San Diego California) for 5 min at 1600 rpm. Cells were then blocked with 2 µL of CD16/32 antibody (Catalog# 101320, BioLegend, San Diego, California) for 10 minutes. An antibody mix was prepared for the surface staining primary antibodies, added to the mixture followed by incubation at 4°C for 30 minutes. Cells were washed, re-suspended in 300 µL of FACS buffer, and analyzed using a BD device (BD LSR Fortessa™, USA), following manufacturer’s instructions. Data was analyzed using Flowjo software (Tree Star, Inc, Ashland, Oregon).

### Statistical analysis

Where appropriate, tests used to determine significance between experiments are outlined in the figure legends of each figure. Data are representative of 2-4 independent experiments for each time points. One way ANOVA was performed using GraphPad Prism7.05 (GraphPad Software, Inc, La Jolla, California) to determine statistical difference. A *P* value ≤0.05 was considered significant.

## Results

### Q-GRFT reduced the fungal burden in a preventive model of murine vaginal candidiasis

**Figure 1:**
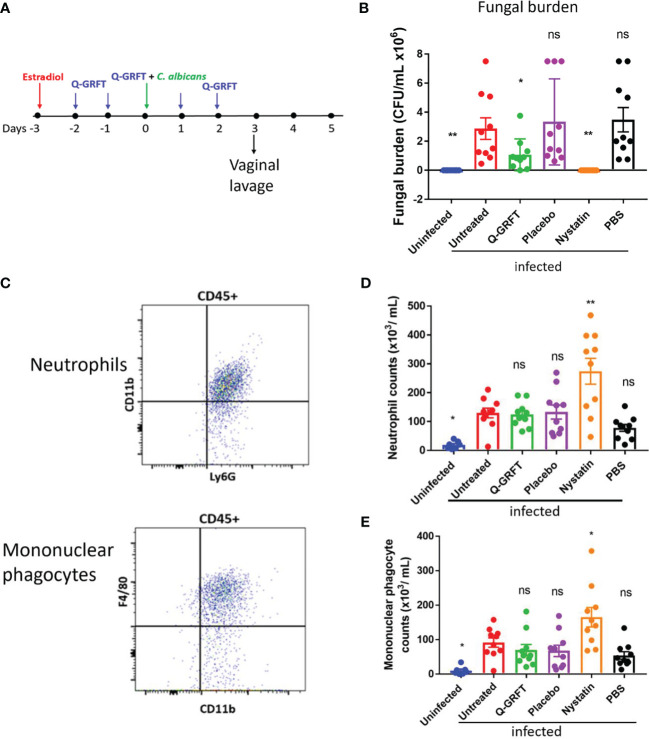
Q-GRFT significantly inhibits vaginal fungal infection in a preventive model of murine candidiasis. **(A)** Experimental scheme. CBA/J mice (N= 10 per group) were estradiol-treated at Day -3, followed by twice daily instillation of either Q-GRFT gel, nystatin solution, PBS, or Carbopol placebo gel per vaginum for the next 5 days. At Day 0, mice were inoculated with 20 µL of **(C)** *albicans* blastospores at a cell concentration of 1.0 X 10^8^ CFU/mL, per vaginum. A vaginal lavage was performed 24 hours after the final dose administration. **(B)** Vaginal fungal burden (CFU/mL) following treatment, for mice in **(A)**. Each dot represents one mouse, N=10 mice per group. Experiments were performed and repeated at least 2 times, and representative data of *Mean ± SEM* is shown. **(C)** Flow cytometry gating strategy for neutrophils, and mononuclear phagocytes in the vaginal lavage. Neutrophils were identified as CD45^+^Ly6G^+^CD11b^+^, while monocytes were CD45^+^CD11^+^F4/80^+^ cells. **(D)** Neutrophil and **(E)** mononuclear phagocyte cell populations in the vaginal lavage following the respective treatments. N=10 animals per group, and each dot represents a population of cells from a single mouse. Measurements are representative of cell populations from experiments performed at least 2 times. *Mean ± SEM* data is presented. For all experiments*, one-way ANOVA* was used for statistical analyses, and *P ≤* 0.05 was considered significant. For normality testing, *Dunnet’s test* **(B)**, and *Tukey’s test* **(D, E)** were performed for respective datasets. For all datasets, all groups were compared to infected untreated controls. 'ns' = p> 0.05; *p=or < 0.05 and '**' = P< or = 0.01.

To evaluate the efficacy of Q-GRFT in a preventive murine model, we established an experimental model for vaginal infection (**Figure 1A**), based on that described by Conti et al. (2014). Female CBA/J mice were injected subcutaneously with estradiol, followed by twice daily vaginal instillation of a Carbopol gel formulation similar to a product that we previously demonstrated had HSV-2 inhibitory activity (Nixon et al., 2013). The Carbopol gel formulation delivered 400 ng Q-GRFT per dose twice daily for 5 days. We challenged the animals with *C. albicans* on day 3. Vaginal lavage was performed 24 hours following administration of the final Q-GRFT treatment. We determined the efficacy of Q-GRFT in the prevention of vaginal candidiasis by establishing the fungal burden in vaginal lavage fluids after vaginal pre-treatment, fungal inoculation, and follow-up treatment with Q-GRFT. Fungal burden was evaluated by plating lavage fluids on Sabourand agar plates that were incubated for 48 hours at 30°C, followed by counting of colonies. Our results demonstrated that Q-GRFT treatment resulted in a significantly lower fungal burden when compared with the infected untreated controls (*P=*0.0417) (**Figure 1B**). Similarly, treatment with the positive control nystatin, a polyene antifungal agent, resulted in a significantly lower fungal burden (*P=*0.0016), while there was no inhibition demonstrated with PBS (*P=*0.4849) and placebo (*P=*0.5963) when compared with the infected controls. Additionally, uninfected animals did not demonstrate any fungal growth (*P=*0.0016).

Upon infection and epithelial penetration, tissue resident-macrophages are among the initial immune cells that encounter *Candida*, and phagocytose the fungal cells to achieve clearance (Netea et al., 2008). Furthermore, pro-inflammatory cytokines released by macrophages and epithelial cells recruit neutrophils and inflammatory monocytes to eradicate *Candida* infection (Netea et al., 2008; Netea et al., 2015). However, recent findings have suggested that while PMNs are generally protective against *C. albicans* at other body sites, they do not appear to be protective in the vagina. Depletion of PMNs using an anti-Ly6G antibody was shown not to impact fungal burden (Peters et al., 2014). Therefore, using flow cytometry, we sought to determine if pre-treatment with Q-GRFT influenced the expression of vaginal innate immune cells [neutrophils (CD45^+^,Ly6G^+^,CD11b^+^) (**Figure 1C** top) and mononuclear phagocytes (CD45^+^CD11^+^F4/80^+^) (**Figure 1C** bottom) in vaginal infection. Compared to infected controls, there was no difference in the population of neutrophils following pre-treatment with either Q-GRFT (*P>*0.9999), placebo (*P>*0.9999), or PBS (*P>*0.6510) (**Figure 1D**). This is consistent with reported observations that PMNs do not contribute to clearance during VVC (Yano et al., 2017). In addition, treatment with nystatin resulted in significantly higher populations of neutrophils (*P=*0.0011), while uninfected animals had lower neutrophils (*P=*0.0483), in comparison with infected controls. Similarly, there was no difference in mononuclear phagocyte populations following treatment with Q-GRFT (*P=*0.9461), placebo (*P=*0.9155), and PBS (*P=*0.6263), in comparison with the infected controls (**Figure 1E**). Nystatin treated animals were associated with higher monocyte populations (*P=*0.0380), while uninfected animals demonstrated significantly lower monocytes (*P=*0.0368) than infected animals. These results demonstrate that while Q-GRFT significantly inhibits *Candida* growth in a preventative murine model, this effect is likely independent of the inflammatory immune response.

### Q-GRFT enhanced clearance of vaginal candidiasis in a therapeutic murine model

**Figure 2:**
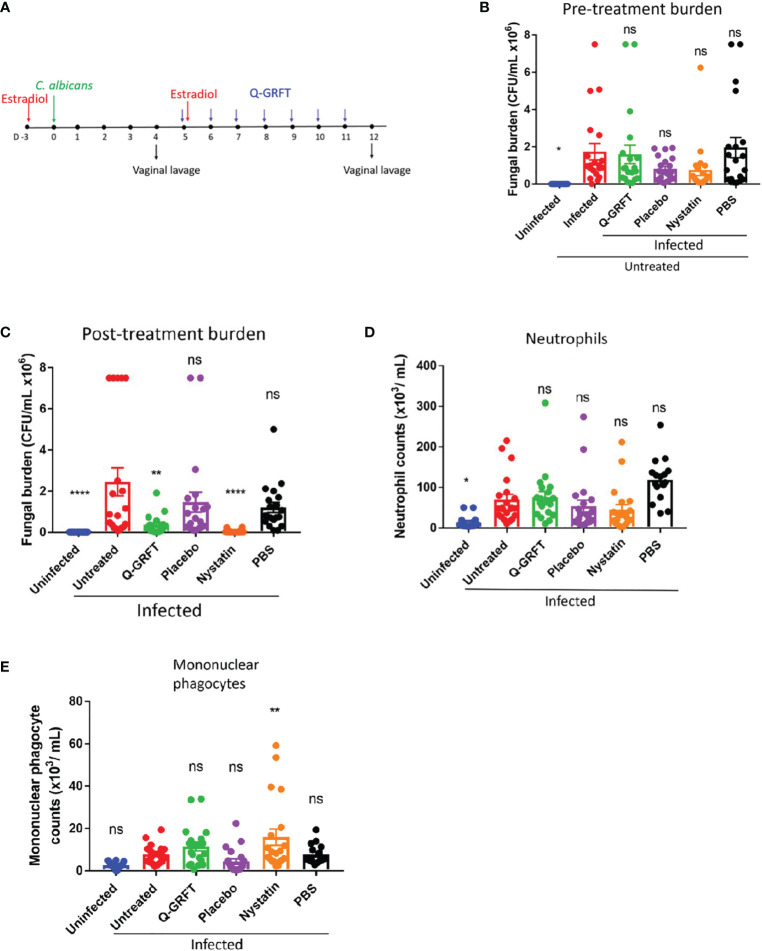
Efficacy of Q-GRFT in a murine model of vaginal candidiasis. **(A)** Experimental scheme. CBA/J mice were estradiol-treated, followed by vaginal inoculation with 20 µL of *C. albicans* blastospores at a cell concentration of 1.0 X 10^8^ CFU/mL 3 days later. Treatment with either Q-GRFT, nystatin, placebo, or PBS by vaginal instillation was started on Day 5 following inoculation and continued twice daily for a total of 7 days, respectively. Vaginal lavage was performed at Day 4 and Day 12 to establish pre-treatment and post-treatment fungal burden, respectively. **(B)** Day 4 (pre-treatment) Vaginal fungal burden (CFU/mL), and **(C)** Day 12 (post-treatment) burden. **(D)** Neutrophil and **(E)** mononuclear phagocyte cell populations in the vaginal lavage following infection and respective treatments, as determined using Flow cytometry. Each dot represents one mouse, N=20 mice per group. Experiments were performed at least 2-3 times and representative data from 2 experiments, *Mean ± SEM* is shown. *One-way ANOVA* was used for statistical analyses and *P ≤* 0.05 was considered significant. For normality testing, *Dunnet’s test* **(B)**, and *Tukey’s test* **(C-E)** were performed for respective datasets. For all datasets, all groups were compared to infected untreated controls. *p=or < 0.05 , ** p=or < 0.01 , ****p =or < 0.0001 and ns=not significant.

To study the role of Q-GRFT in the treatment of candidiasis, a murine therapeutic experimental model was developed, (**Figure 2A**), based on that described by Conti et al. (2014). Mice were injected subcutaneously with estradiol, followed by inoculation with *C. albicans* vaginally 3 days later. Vaginal lavage was performed on day 4 following fungal challenge, to determine baseline fungal burden. Twice daily vaginal instillation of 400 ng Q-GRFT was started on day 5 and continued for a total of 7 days. A vaginal lavage was performed 24 hours after the final dose to determine fungal burden by colony counts on Sabourand agar plates, and immune response to treatment using flow cytometry. Pre-treatment fungal burden (**Figure 2B**) confirmed that all mice had established vaginal infection prior to initiating treatment, with no significant differences in fungal burden in any of the infected groups prior to initiation of treatment. Compared to placebo, treatment with topical vaginal Q-GRFT gel resulted in a significant inhibition of *C. albicans* burden (*P=*0.0379), similar to that seen with the control nystatin (*P=*0.0003), at the end of the dosing period (**Figure 2C**). These results indicated that Q-GRFT was an effective treatment for vaginal candidiasis in a murine model.

### Treatment with Q-GRFT does not induce overt changes in innate immune cell phenotypes in vaginal candidiasis

Pro-inflammatory cytokines released by macrophages and epithelial cells recruit neutrophils and inflammatory monocytes during *Candida* infection (Netea et al., 2008; Netea et al., 2015). Therefore, we next sought to determine if treatment with Q-GRFT influenced the expression of vaginal innate immune cells, neutrophils (CD45^+^Ly6G^+^CD11b^+^), (**Figure 2D**), and mononuclear phagocytes (CD45^+^CD11b^+^F4/80^+^), (**Figure 2E**), in candidiasis using flow cytometry. Compared to infected controls, Q-GRFT did not induce any significant changes in populations of both neutrophils, *P=*0.7279, and mononuclear phagocytes, *P=*0.1960. Similarly, neutrophils populations were not significantly different between infected controls and nystatin treated mice, *P=*0.1771, while monocytes were elevated following treatment, *P=*0.0055. Compared with the infected untreated controls, uninfected mice demonstrated significantly lower neutrophils (*P=*0.0039), but not mononuclear phagocytes, *P=*0.0873. Both placebo and PBS did not result in any changes in neutrophil (*P=*0.3626, *P=*0.111), and monocyte (*P=*0.2464, *P=*0.9939) populations, respectively, when compared to infected untreated animals. These results demonstrate that Q-GRFT does not induce overt changes in neutrophil and mononuclear phagocytic populations following vaginal infection with *C. albicans*.

### Histology of vaginal tissue following treatment with Q-GRFT demonstrates paucity of infection

**Figure 3:**
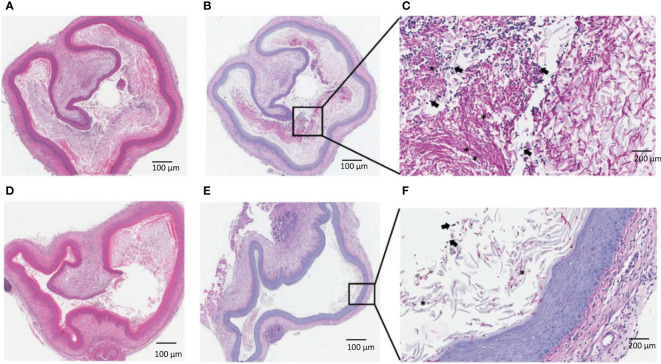
Histological evaluation of vaginal candidiasis following treatment with Q-GRFT. CBA/J mice were infected with *C. albicans* vaginally. 5 days later, topical treatment was initiated with Q-GRFT gel (vaginally) twice daily for 7 days. Post-treatment (Day 12) tissue histology is presented. **(A)** Representative H&E staining of mice vaginal tissue in the infected, untreated group. **(B, C)** PAS staining of infected, untreated mice. Note the predominance of fungal growth/burden in the vaginal lumen. **(D)** Representative H&E staining of vagina tissues from Q-GRFT treated mice. **(E, F)**, PAS staining of vaginal tissue from Q-GRFT-treated mice. (*) represents fungal hyphae and black arrows depict neutrophils in the vagina.

To further investigate the effect of Q-GRFT on vaginal candidiasis, we evaluated the impact of topical administration on the histology of infected tissues at the end of the drug treatment period. Microscopic analysis revealed that infected untreated animals displayed significant vaginal luminal congestion with high fungal growth/burden (**Figures 3A-C**), unlike Q-GRFT-treated animals that displayed lower congestion (**Figures 3D-F**). Consistent with our microbiological observations, H&E and PAS staining demonstrated that Q-GRFT was an effective treatment against treated vaginal candidiasis.

## Discussion

Our study demonstrated that Q-GRFT significantly inhibited vaginal infection in a preventive model and enhanced fungal clearance in a therapeutic murine model of candidiasis. Cytokines expressed in the vagina following *C. albicans* infection induce large populations of neutrophils in the epithelium, underscoring their mucosal immune cell activation and recruitment function (Zhang et al., 2018). Recent findings, however, suggest that neutrophils recruited into the vagina during VCC are unable to clear fungal infection due to a combination of numerous factors in the local milieu. First, the protective role of PMNs appears to be impeded by heparan sulfate in VVC that induces their dysfunction (Yano et al., 2017). Heparan sulfate is a competitive ligand for the neutrophilic receptor Mac-1, yet the receptor is critical in fungal recognition and neutrophil-mediated killing (Ardizzoni et al., 2021). Additionally, women with symptomatic VVC have demonstrated a higher frequency of anti-*C. albicans* antibodies, CAGTA and perinuclear anti-neutrophils cytoplasmic antibodies (pANCA) than healthy controls (Ardizzoni et al., 2021). *In vitro* studies have shown that pANCA completely blocks the *Candida* killing activity of neutrophils freshly isolated from healthy donors (Ardizzoni et al., 2021). Interestingly, Zhang et al. showed that epithelial treatment with nystatin, further enhanced the initial immune process generated early in infection, resulting in fungal clearance (353). Although fewer in number, macrophages may be recruited into vaginal tissues during infection, and act as antigen presenting cells when activated, generating pro-inflammatory and cytotoxic T cell responses (Wira et al., 2005; Iijima et al., 2008; Hickey et al., 2011; Kalia et al., 2019). In our preventive model, we have demonstrated that pre-treatment with Q-GRFT prevented infection with *C. albicans* but did not affect the frequency of neutrophil and mononuclear phagocytes populations, in comparison with infected control animals. In the therapeutic model, unlike the infected controls, treatment with Q-GRFT was effective in fungal clearance with no demonstrable differences in local neutrophil and mononuclear phagocytic populations. These results are similar to observations in the nystatin treatment animals. Given the data suggesting anergy of innate immune cells in VVC (Yano et al., 2017; Ardizzoni et al., 2021), our results demonstrate that Q-GRFT enhances fungal clearance, and the inhibitory role is likely independent of the local inflammatory response.

Human live vaginal challenge studies have demonstrated that protection from candidiasis is associated with asymptomatic colonization with *Candida*, and the absence of any inflammatory response. Additionally, a heavy inflammatory response with cellular predominance of PMNs is observed in symptomatic disease (Yano et al., 2012). Furthermore, a positive correlation has been observed between PMN infiltration and vaginal fungal burden in a subset of individuals (Fidel et al., 2004). Comparably, in murine studies, a heavy vaginal infiltration with PMNs has been observed in subsets of inoculated animals, despite no impact on the fungal burden (Fidel et al., 1996; Yano et al., 2010). Given the failure in characterizing disease severity in murine models based on clinical signs and symptoms of vaginitis, the rigid criteria based on high and low PMN responses ably predicts symptomatic and asymptomatic conditions in mice with VVC (Yano et al., 2010; Yano et al., 2012). In fact, *in vitro* PMN migration assays have demonstrated that vaginal lavage fluids from high PMN (symptomatic) mice have higher chemotactic activity, when compared to those from low PMN (asymptomatic) animals (Yano et al., 2010).

In extensive *in vitro* and *in vivo* assays and experimental models, secretion of cytokines and chemokines by epithelial cells and tissues is only minimally changed upon treatment with Griffithsin (O'Keefe et al., 2009; Kouokam et al., 2011; Kouokam et al., 2016). The lack of difference in populations of neutrophils and monocytes triggered following vaginal infection in both Q-GRFT treated and untreated animals is indicative of a likely direct inhibitory role of the lectin against *Candida*.

Infection with *C. albicans* likely triggered cytokines early in infection in the therapeutic model, attracting innate immune cells among all mice inoculated with fungal blastospores. Murine treatment with Q-GRFT, as well as in the control animals with nystatin resulted in fungal clearance, albeit with a detectably low fungal burden upon completion of the dosing period. In the preventive model, inflammatory infiltrates (neutrophils and mononuclear phagocytes) were still elevated at the end of the study period, similar to therapeutic animals, in both Q-GRFT and nystatin treated animals. It is unlikely that Q-GRFT induced inflammatory infiltrates in the lectin treated groups, given our prior comprehensive studies that demonstrated the lack of immune stimulation by GRFT when applied in the vagina of experimental animals (O'Keefe et al., 2009; Kouokam et al., 2016). However, in murine vaginal experiments, nystatin enhances the immune inflammatory response against *C. albicans* (Zhang et al., 2018), which likely accounts for the persistent cellular infiltrates observed in treated animals at the end of the study period. The absence of immune stimulation by Q-GRFT is likely advantageous to its potential utility in multipurpose microbicide technologies as an antiviral and antifungal product. HIV-1 transmission is enhanced in the presence of inflammation (Lavelle et al., 2010; Passmore et al., 2016), hence utility of Q-GRFT to prevent vaginal candidiasis is not likely to increase the risk of infection.

There is scant data available assessing the impact of plant-derived lectins in VVC. Bruno Severo Gomes and colleagues have described the antifungal activity of lectins *Dioclea violacea* (Dviol), *D. rostrata* (DRL) and *Canavalia brasiliensis* (ConBr) against yeast isolated from vaginal secretions using *in vitro* assays (Gomes et al., 2012). Although the exact mechanism of action of these lectins is not identified, they postulate that it is likely a direct inhibitory effect involving alteration of the fungal cell wall and other synthesis pathways (Gomes et al., 2012). Similarly, our earlier *in vitro* studies identified α-mannan in the *C. albicans* cell wall as a binding target for Q-GRFT (Nabeta et al., 2021). Additionally, we demonstrated that Q-GRFT’s inhibitory activity involved the differential expression of genes involved in stress response and cell cycle regulation (Nabeta et al., 2021). Q-GRFT binds to α-mannan, and disrupts cell wall and membrane integrity, causing desiccation and loss of fungal budding ability. We assume that the mechanism of action *in vivo* is related to this *in vitro* observation. Altogether, the *in vitro* findings (Nabeta et al., 2021), and murine results demonstrating a lower fungal burden with Q-GRFT treatment, suggest a direct inhibitory impact against *C. albicans*.

So far, there is no demonstrated toxicity, T-cell activation, or immunological stimulation of GRFT or Q-GRFT in *in vitro* and *in vivo* studies (Kouokam et al., 2011; Kouokam et al., 2016; Girard et al., 2018). Here, we have demonstrated that Q-GRFT significantly inhibited infection in a preventive model, and enhanced candidiasis clearance in murine therapeutic studies. Altogether, these data suggest that Q-GRFT likely directly inhibits vaginal *C. albicans* growth, regardless of the inflammatory status in the local milieu. In this study, we did not characterize cytokines expression in both preventive and therapeutic model, despite demonstrating fungal clearance with Q-GRFT treatment. This limitation warrants further exploration to gain a deeper understanding of the role Q-GRFT plays in VVC. Additionally, assessing the neutrophilic and monocytes phagocytic killing activity in the presence of Q-GRFT will provide a better understanding of Q-GRFT’s role in VVC. Q-GRFT has shown promise in preventing viral sexually transmitted infections, including HSV-2 and HIV-1 (Nixon et al., 2013; Derby et al., 2018; Tyo et al., 2020; Tyo et al., 2020). Our data demonstrate additional potential for utility of Q-GRFT vaginal dosage forms in both preventing and treating candidiasis, and further support incorporation of Q-GRFT in multipurpose STI prevention modalities.

## Data availability statement

The raw data supporting the conclusions of this article will be made available by the authors, without undue reservation.

## Ethics statement

The animal study was reviewed and approved by University of Louisville Institutional Animal Care and Use Committee.

## Author contributions

HN and KP conceived and designed the experiments. NHWperformed the experiments. HN, AL, and KP analyzed the data. LW and LR designed the Carbopol-based gel formulation and manufactured Q-GRFT product that was tested in these studies. HN, JF and KP further contributed reagents and materials tools. HN and KP wrote the paper. All authors contributed to the article and approved the submitted version.

## Funding

This work was supported by a grant from ExCITE/NIH U01HL127518 to HN and NIH/NIAID 5U19AI113182 to KP. The contents of this work are solely the responsibility of the authors and do not represent the official views of the NIH.

## Conflict of interest

The University of Louisville Research Foundation has filed patent applications claiming the use of Griffithsin in preventing and treating fungal infections. HN and KP are named as inventors.

The remaining authors declare that the research was conducted in the absence of any commercial or financial relationships that could be construed as a potential conflict of interest.

## Publisher’s note

All claims expressed in this article are solely those of the authors and do not necessarily represent those of their affiliated organizations, or those of the publisher, the editors and the reviewers. Any product that may be evaluated in this article, or claim that may be made by its manufacturer, is not guaranteed or endorsed by the publisher.
